# Evaluation of quality of life in adults with neurofibromatosis 1 (NF1) using the Impact of NF1 on Quality Of Life (INF1-QOL) questionnaire

**DOI:** 10.1186/s12955-017-0607-y

**Published:** 2017-02-14

**Authors:** Rosalie E Ferner, Mary Thomas, Gemma Mercer, Victoria Williams, Guy D Leschziner, Shazia K Afridi, John F Golding

**Affiliations:** 1 National Neurofibromatosis Service, Department of Neurology, Guy’s and St. Thomas’ NHS Foundation Trust, Great Maze Pond, London, SE1 9RT UK; 2 National Neurofibromatosis Service, Department of Genetic Medicine St. Mary’s Hospital, Manchester, UK; 3 Department of Psychology, University of Westminster, London, UK; 4 Department of Clinical Neuroscience, Intistute of Psychiatry, Psychology & Neuroscience, King’s College London, Great Maze Pond, London, SE1 9RT UK

## Abstract

**Background:**

Neurofibromatosis 1 (NF1) is an inherited, multi-system, tumour suppressor disorder with variable complications that cause psychological distress and social isolation. The study aim was to develop and validate a disease-specific questionnaire to measure quality of life (QOL) in NF1 that is suitable both as an assessment tool in clinical practice and in clinical trials of novel therapy.

**Methods:**

The Impact of NF1 on Quality of Life (INF1-QOL) questionnaire was developed by a literature search for common terms, focus group (*n* = 6), semi-structured interviews (*n* = 21), initial drafts (*n* =50) and final 14 item questionnaire (*n* = 50). Bivariate correlations between items, exploratory factor analysis, correlations with severity and EuroQol were employed.

**Results:**

INF1-QOL showed good internal reliability (Cronbach’s alpha 0.87), mean total INF1-QOL score was 8.64 (SD 6.3), median 7.00, range 0–30 (possible range 0–42); no significant correlations with age or gender. The mean total EuroQol score was 7.38 (SD 2.87), median 6.5, mean global EuroQol score was 76.34 (SD 16.56), median 80. Total INF1-QOL score correlated with total EuroQol *r* = 0.82, *p* < 0.0001. The highest impact on QOL was moderate or severe problems with anxiety and depression (32%) and negative effects of NF1 on role and outlook on life (42%). The mean inter-relater reliability for grading of clinical severity scores was 0.71 (range 0.65-0.79), and intra-class correlation was 0.92. The mean clinical severity score was 1.95 (SD 0.65) correlating *r* = 0.34 with total INF1-QOL score *p* < 0.05 and correlated 0.37 with total EuroQol score *p* < 0.01. The clinical severity score was mild in 17 (34%), moderate in 16 (32%) and 17 (34%) individuals had severe disease.

**Conclusions:**

INF1-QOL is a validated, reliable disease specific questionnaire that is easy and quick to complete. Role and outlook on life and anxiety and depression have the highest impact on QOL indicating the variability, severity and unpredictability of NF1. INFI-QOL correlates moderately with clinical severity. The moderate relationship between INF1-QOL and physician rated severity emphasizes the difference between clinical and patient perception. INFI-QOL will be useful in individual patient assessment and as an outcome measure for clinical trials.

**Electronic supplementary material:**

The online version of this article (doi:10.1186/s12955-017-0607-y) contains supplementary material, which is available to authorized users.

## Background

Neurofibromatosis 1 (NF1) is a common, autosomal dominantly inherited disease that primarily involves the nervous system, eye, skin, and bone [1]. NF1 is associated with an increased frequency of benign and cancerous tumours and the hallmark lesion is the neurofibroma, a benign peripheral nerve sheath tumour. The complications are extensive and range from learning difficulties, central nervous system tumours, neurovascular disease, sleep disturbance, hypertension and scoliosis, to neurofibromas causing disfigurement, nerve root and spinal cord compression and malignancy [1]. The clinical manifestations are variable, unpredictable and potentially life threatening. Disfigurement and social isolation represent potent causes of psychological distress and there is a 50% risk of passing on NF1 to an offspring [1, 2].

Advances in molecular biology have facilitated the development of novel therapy including drugs that have the potential to treat symptomatic neurofibromas. A phase 1 study of selumetinib was performed in NF1 children and in young adults after preclinical research showed a reduction in size of plexiform neurofibromas with mitogen activated protein kinase kinase pathway (MEK) inhibition [3]. Recent results suggest that selumetinib is helpful in treating inoperable, symptomatic plexiform neurofibromas [4, 5]. Robust outcome measures are essential to evaluate the efficacy of the therapy, from both the clinician’s and the patient’s standpoint. Current practice is to measure disease progression in NF1 by clinical and neurological assessment, magnetic resonance imaging and positron emission tomography [1].

The impact of NF1 on the individual’s quality of life (QOL) is more difficult to evaluate and there is a need for disease-specific questionnaires in NF1 that will evaluate health related QOL. QOL is recognised as an important marker of disease progression, and as an outcome measure following intervention. QOL may be assessed using semi-structured interviews, generic or disease-specific questionnaires. Semi-structured interviews were performed in Australian NF1 adults with varying disease severity and visible neurofibromas [6]. The participants experienced psychological distress related to learning problems, pain and the cosmetic burden of NF1. They were concerned about the uncertainty of disease progression and the risk of passing on NF1 to their offspring. A Brazilian study of NF1 adults used the generic WHOQOL-100 and semi-structured interviews [7]. Patients were apprehensive about disease visibility affecting social relationships. Confusion about distinguishing NF1 from contagious diseases and disease variability were also distressing. Mautner et al. undertook a variety of physical assessments and generic psychosocial measures to assess quality of life in NF1 individuals [8]. They reported that patients had a negative body image that resulted in low self-confidence and psychological distress.

Generic questionnaires such as the Short-Form 36 (SF-36) and EuroQol are widely used in clinical practice and research [9, 10}. However, the SF-36 is lengthy, which is problematic in NF1 patients who have impaired sustained attention [1]. Neither of these questionnaires [9, 10] specifically addresses symptoms related to NF1. The Skindex questionnaire is useful to evaluate the impact of the skin manifestations of NF1 on QOL but does not deal with the other significant complications associated with the disease [11]. Wolkenstein et al. administered the SF-36 and Skindex questionnaires to NF1 patients and noted that more visible disease had a greater impact on physical function, pain, general health and vitality [11].

At present there is only one disease-specific QOL questionnaire for NF1 adults reflecting quantitative assessment of quality of life [12]. The adult version of PedsQL™ was developed as a module for NF1 adults from PedsQL™ and integrates generic and disease specific questions. The adult PedsQL™ has multiple physical, social, emotional, cognitive domains and assessments of daily functioning. However, only 15 adults participated in the initial development of the questionnaire and there was no independent clinician rating of physical severity. The self-report instrument comprises 70 items, but omits evaluation of important problems in NF1, including Bone Health and Sleep disorders. The large number of questions increases the risk of missed or inaccurate responses, particularly in patients with cognitive problems.

Previously, we have worked with patients and clinicians to devise a validated, disease-specific questionnaire for neurofibromatosis 2 (NF2), a rare tumour suppressor condition, characterised by bilateral vestibular schwannomas and other nervous system tumours [13, 14]. The questionnaire is simple to complete, correlates well with clinician-related severity and has been adopted widely by clinicians to assess quality of life in NF2.

### Aims

The aim of this study was to develop and validate a disease-specific questionnaire to measure QOL in people with NF1, that is suitable as an assessment tool in clinical practice and in clinical trials, and that is quick and simple to complete. Furthermore, the goal was to assess the impact of diagnosis, management and burden of disease on the individual with NF1.

## Methods

Guy’s and St. Thomas’ NHS Foundation Trust (GSTT) and Central Manchester University Hospitals Foundation Trust (CMFT) are national centres for the diagnosis, management and support of approximately 1000 people with mild and severe NF1 in each centre. Adults with NF1 were approached when they attended the clinic and given information sheets identifying the aims of the projects and inviting them to participate in the study. Adults (18 years or older) who fulfilled the diagnostic criteria for NF1 (NIH Consensus Development Conference 1987) [15] and attended the National NF1 service at GSTT or CMFT were included. NF1 individuals under 18 years, people who did not fulfil the diagnostic criteria for NF1 and patients who were unable to give informed consent were excluded. Interpreters were available and support was offered by the clinical nurse specialist to patients with cognitive and literacy problems, visual impairment, upper limb weakness, numbness or incoordination.

Ethical Committee approval for the study was obtained from County Durham and Tees Valley Research and Ethics Committee. Written informed consent was obtained from every participant.

Disease severity was determined using the Riccardi NF severity grade classification 1 to 4 [16]. Grades 1 and 2 were amalgamated to ensure adequate numbers in each group. Grades 1 and 2 were deemed mild, grade 3 moderate and grade 4 severe. Four clinicians (three neurology consultants and a clinical nurse specialist) with expertise in NF1 rated the patient clinical problem list independently.

### Development of INF1-QOL questionnaire

The impact of neurofibromatosis one on quality of life questionnaire was named INF1-QOL and the questionnaire was developed using sequential stages to ensure robust construction. A comprehensive list of symptoms, social and emotional difficulties related to NF1 was produced using a literature review, qualitative interviews and advice from clinicians and nurses with expertise in NF1.

As part of routine care, six sequential patients attending the NF1 service were invited to a focus group session to discuss symptoms and concerns that could impact on NF1 QOL. The session was manually recorded and coded and identifying data were removed. GSTT and CMFT recruited 21 individuals to participate in qualitative interviews and they were representative of the general NF1 population as regards age, gender and disease severity. The in-depth interview was semi-structured and conducted in a secure private environment [17]. All interviews were recorded and transcribed, identifying data were removed and analysis was carried out using a framework approach [17].

A list of 55 items was generated by these processes and analysed by a multi-disciplinary panel of NF1 specialists and a psychologist to produce a pilot questionnaire using the previously proven systematic approach [13]. The 16 item pilot questionnaire was completed by 50 NF1 participants.

The pilot questionnaire was analysed by the following: bivariate correlations between items, exploratory factor analysis and correlations with physician severity scoring. The pilot questionnaire comprised 16 items that the participant rated as no problem, mild, moderate or severe. After completion of the 16 item pilot questionnaire, the questions on interactions with health services and other people’s attitudes to NF1 were removed due to redundancy with other items.

The final questionnaire was completed by a further 50 patients. It comprised 14 items that were rated by the participant in the same way as the pilot questionnaire. The maximum potential score for the 14 item questionnaire was 42, each item was scored from zero to three, and the highest score denoted the worst function. A free text section was included at the end for participants to provide an explanation and expanded information on any item that impacted on QOL.

The final 14 item INF1-QOL questionnaire was administered with a generic measure of QOL. The EuroQol questionnaires were completed by the 50 NF1 participants who did the final 14 item INF1-QOL questionnaire. The EuroQol (EQ 5D) contains a global health score and covers Mobility, Self-care, Usual Activities, Pain/Discomfort, Anxiety and Depression [10]. Physician rated severity score and EuroQol provided some estimate of external validity in the 14 item pilot questionnaire.

Participants were offered long-term follow-up in GSTT or CMFT national NF1 centres as part of routine clinical care for people with neurofibromatosis 1.

## Results

Six individuals attended a focus group, 21 people participated in the semi-structured interviews, 50 completed the first version of the questionnaire (16 items) and a further 50 patients completed the final version (14 items) and the EuroQol. Physician rated severity scores were available for each stage of testing. The participants included 44% males and 56% females, the mean age was 38.3 years (SD 14.1) and the range was 18–77 years.

Fifty five items were generated from the literature search, the NF1 specialists, the patient focus group and the qualitative interviews. The items were grouped into eight themes including school difficulties, work issues, physical problems, emotions and feelings, activities of daily living and leisure activities, interaction with health services, other people’s attitude to NF1, and relationships with employers, family and or friends. The 16 item pilot questionnaire was constructed to reflect these themes and after completion interactions with health services and other people’s attitudes to NF1 were removed due to redundancy with other items. The final questionnaire comprised 14 items and a free text section was provided at the end.

The final version included the following items reflecting the six remaining themes: Vision, Cosmetic appearance, Pain (quality and intensity), Learning Problems, Behaviour and Personality, Mobility and Walking, Hand Function, Speech, Bone Health, Breathing, Sleeping, Role and Outlook on Life, Depression and Anxiety (Additional file 1).

**Fig. 1:**
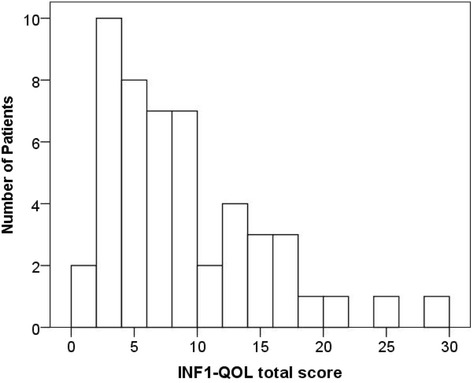
Total INF1-QOL score distribution in 50 NF1 participants

The final version of the INF1-QOL questionnaire showed good internal reliability (Cronbach’s alpha 0.87). The mean total INF1-QOL score was 8.64 (SD 6.3), median 7.0 with a range of 0–30 (Fig. 1) and there was no significant correlation with age or gender. The individual item responses are shown in Table 1, detailing the number and percentage of individuals with no problem, mild, moderate or severe problems for each of the 14 questions. The mean total EuroQol score was 7.38 (SD 2.87), median 6.5, with an observed range of 5–18 and the mean global EuroQol score was 76.34 (SD 16.56), median 80, range of 25–100. The correlation of total INF1-QOL score with total EuroQol was *r* = 0.82, *p* < 0.0001. The individual item responses for the five questions (no problem, slight, moderate, severe or extreme problems) are shown in Table 2.

**Table 1:** The INF1-QOL 14 item questionnaire – responses in 50 NF1 participants

INF1-QOL Question	No problem n (%)	Mild Problem n (%)	Moderate Problem n (%)	Severe Problem n (%)	Pearson Correlation with Total INF1-QOL Score (p 2-tailed)
Q1 Vision	24 (48)	20 (40)	5 (10)	1 (2)	.380 (.006)
Q2 Cosmetic appearance	26 (52)	16 (32)	4 (8)	4 (8)	.623 (<.001)
Q3 Pain quality	25 (50)	20 (40)	4 (8)	1 (2)	.810 (<.001)
Q4 Pain Intensity	21 (42)	19 (38)	8 (16)	2 (4)	.671 (<.001)
Q5 Learning problems	16 (32)	25 (50)	9 (18)	0 (0)	.678 (<.001)
Q6 Behaviour and personality	27 (54)	18 (36)	4 (8)	1 (2)	.569 (<.001)
Q7 Mobility and walking	42 (84)	4 (8)	4 (8)	0 (0)	.620 (<.001)
Q8 Weakness, numbness, clumsiness in hands	37 (72)	11 (24)	2 (4)	0 (0)	.698 (<.001)
Q9 Speech	36 (72)	12 (24)	2 (4)	0 (0)	.496 (<.001)
Q10 Bones	37 (72)	11 (24)	2 (4)	0 (0)	.608 (<.001)
Q11 Breathing	42 (84)	8 (16)	0 (0)	0 (0)	.351 (.012)
Q12 Sleeping	22 (44)	19 (38)	8 (16)	1 (2)	.598 (<.001)
Q13 Role and outlook on life	13 (26)	16 (32)	14 (28)	7 (14)	.693 (<.001)
Q14 Depression and anxiety	18 (36)	16 (32)	14 (28)	2 (4)	.802 (<.001)

**Table 2:** European Quality of Life (EQ-5D-5 L) Health Questionnaire – responses in 50 NF1 participants

Questions	No problem n (%)	Slight problems n (%)	Moderate problems n (%)	Severe problems n (%)	Extreme problems n (%)
Q1 Mobility	40 (80)	8 (16)	1 (2)	1 (2)	0 (0)
Q2 Self-Care	46 (92)	1 (2)	3 (6)	0 (0)	0 (0)
Q3 Usual Activities	37 (74)	7 (14)	4 (8)	1 (2)	1 (2)
Q4 Pain/Discomfort	24 (48)	20 (40)	6 (12)	0 (0)	0 (0)
Q5 Anxiety/Depression	24 (48)	13 (26)	8 (16)	4 (8)	1 (2)

The mean inter-relater reliability for the grading of clinical severity scores was 0.71 (range 0.65-0.79), and the intra-class correlation was 0.92. The mean clinical severity score was 1.95 (SD 0.65) correlated *r* = 0.34 with total INF1-QOL score *p* < 0.05 and correlated 0.37 with total EuroQol score *p* < 0.01. The clinical severity score was averaged across raters and 17 (34%) patients had mild NF1, 16 (32%) had moderate disease and 17 (34%) were rated as severe.

## Discussion

We have developed a disease specific quality of life questionnaire that encompasses the wide variation in phenotype in NF1 adults. All the participants reported that INF1-QOL was easy to understand and were able to finish it within ten minutes. INF1-QOL correlated highly with EuroQol; although EuroQol was quicker to complete, the INF1-QOL had a broader range of themes and was disease specific. Moreover, the space for free text permitted the patient to clarify an answer and to help us determine whether the problem was related to NF1. For instance, visual impairment was due to refraction problems in one individual and not caused by a co-existing NF1 related optic pathway glioma. The free text also provided the opportunity to highlight personal issues that a patient might find difficult to discuss within a multi-disciplinary clinic.

The highest recorded score was 30 out of a possible total of 42 and implies that the impact of the disease on quality of life may be severe in some but not all items. This emphasizes the broad range and variability of the NF1 phenotype. Anxiety and depression and role and outlook on life had the highest impact on QOL. There were moderate or severe problems in 32% of participants with anxiety and depression and moderate or severe problems due to effect of NF1 on role and outlook on life in 42% of individuals. This is readily explained by a chronic, inherited and unpredictable disease where illness adjustment is a significant feature.

Pain quality correlated with total INF1-QOL score rather more than pain intensity but both have a major impact on quality of life in people with NF1. Bone dysplasia, benign and malignant peripheral nerve sheath tumours may cause significant pain and pain intensity can be helpful in distinguishing benign neurofibromas from cancerous tumours [1]. Cosmetic problems were rated as mild in 32% and moderate or severe in 16% of patients, highlighting that the visible manifestations of the disease are a potent cause of distress [6–8, 11]. Although participants reported only mild problems in some domains such as breathing, these symptoms still impact significantly on quality of life in people with multiple and unpredictable manifestations of disease.

Importantly, our study was representative of the adult NF1 population as a whole and the patients were well distributed for age and gender. The clinician rated disease severity demonstrated even spread across the three rankings mild, moderate and severe and there was good inter-rater reliability. There was a moderate relationship between clinician rated severity and patient rated quality of life and likely reflects the difference in disease perception between patient and clinician. This distinction is crucial, particularly in the context of evaluating novel therapy. A drug will be of limited benefit, if for instance, it reduces tumour size, but the patient perceives no improvement of quality of life.

## Conclusions

INF1-QOL is a validated, reliable disease specific questionnaire that correlates moderately well with disease severity. The next stage will be to determine the reliability of INFI-QOL over time and potentially the questionnaire could be developed in computerised form. INFI-QOL is simple and quick to complete and covers a broad range of themes, representative of NF1 manifestations. We believe that it will be helpful in monitoring quality of life in the clinic setting as well as a disease outcome measure in clinical trials and therapeutic intervention.

## Additional file

Additional file 1: Impact of neurofibromatosis 1 on quality of life questionnaire (INF1-QOL). (DOCX 34 kb)
