# Supplementary material for: Evaluation of quality of life in adults with neurofibromatosis 1 (NF1) using the Impact of NF1 on Quality Of Life (INF1-QOL) questionnaire
Source: Health Qual Life Outcomes. 2017 Feb 14;15:34. doi: 10.1186/s12955-017-0607-y (PMC5307827; doi:10.1186/s12955-017-0607-y)
Supplement: Additional file 1: — Impact of neurofibromatosis 1 on quality of life questionnaire (INF1-QOL). (DOCX 34 kb) [file 12955_2017_607_MOESM1_ESM.docx]

IMPACT OF NEUROFIBROMATOSIS 1 ON QUALITY OF LIFE QUESTIONNAIRE QOL (INF1-QOL)

Please complete the following information

Name

Hospital ID

Date of Birth

Age in years

Please tick one box below

Gender Male Female

Date questionnaire completed

**For EACH of the questions please tick ONE box that best describes how you have felt in the last month.**

Daily activities e.g. washing, dressing cooking, housework

Social activities e.g. meeting family or friends, going to cinema, pub, cafe, sport

Q1 Do problems with your vision interfere with studying, work, daily living activities, or social activities?

No problems with vision □0

Mild problems but able to perform activities □1

Moderate problems with vision cause me difficulty in performing activities □2

Severe vision problems stop my activities □3

Q2 Does the cosmetic appearance of your neurofibromas (the way they look) interfere with studying, work, daily living activities or social activities?

No difficulties with appearance of my neurofibromas □0

Mild difficulties but able to perform activities □1

The appearance of my neurofibromas causes moderate difficulty

in performing activities □2

The cosmetic appearance of my neurofibromas stops my activities □3

Q3 Pain – Do you suffer from pain that interferes with studying, work, daily living activities or social activities

No problems with pain □0

Mild problems but able to perform activities □1

Pain causes me moderate difficulty in performing activities □2

Severe pain stops my activities □3

Q4 How bad is your pain usually?

0 = no pain □0

1-4 = mild pain □1

5-7 = moderate pain □2

8-10 = severe pain □3

Q5 Do learning problems

(e.g. problems with reading, writing, spelling, maths, concentration, co-ordination, organisation) interfere with studying, work, daily living activities or social activities?

No problems with learning □0

Mild problems but able to perform activities □1

Learning problems cause me moderate difficulty in performing activities □2

Severe learning problems stop my activities □3

Q6 Do you have problems with your behaviour or personality that interfere with your studies, work daily living activities or social activities (e.g. difficulty in making friends, autism, difficulty in understanding jokes, gestures and body language?)

No problems with behaviour or personality □0

Mild problems but able to perform activities □1

Behaviour or personality causes me moderate difficulty in performing activities □2

Severe behaviour or personality problems stop my activities □3

Q7 Do you have problems with mobility and walking?

No problems with mobility and walking □0

Mild problems but can manage without help □1

Moderate problems with walking – need help to walk □2

Severe problems walking -unable to walk at all □3

Q8 Do you have problems with weakness, numbness or clumsiness of your hands that interfere with studying, work, daily living activities or social activities (e.g. using a knife and fork, writing, doing up buttons, doing the cooking, brushing your hair)?

No problems with using my hands □0

Mild problems but can manage without help □1

Moderate problems with using my hands– need help with daily living activities □2

Severe problems with using my hands -unable to do daily living activities □3

Q9 Do problems with your speech interfere with studying, work, daily living activities or social activities?

No problems with speech □0

Mild problems but able to perform activities □1

Moderate speech problems cause me some difficulty

in performing activities □2

Severe speech problems stop my activities □3

Q 10 Do problems with your bones interfere with studying, work, daily living activities or social activities? (e.g. scoliosis or curving of spine; pseudarthrosis – curving and fracture of bones in legs or arms; osteoporosis )

No problems with bones □0

Mild problems with bones but able to carry out activities □1

Moderate problems with bones – need help to carry out daily activities □2

Severe problems with bones stop me carrying out activities □3

Q11 Do problems with your breathing interfere with studying, work, daily living activities or social activities?

No problems with breathing □0

Mild problems with breathing but able to carry out activities □1

Moderate problems with breathing – need help to carry out activities □2

Severe problems with breathing stop me carrying out activities □3

Q12 Do problems with sleeping interfere with studying, work, daily living activities or social activities? (e.g. difficulty getting to sleep, waking up early or during the night, restless sleeping, bad dreams, excessive sleepiness)

No problems with sleep □0

Mild problems but able to perform activities □1

Moderate sleep problems cause me some difficulty in performing activities □2

Severe sleep problems stop my activities □3

Q13 Has NF1 affected your role and outlook on life? (e.g. career, confidence, relationships, caring for family, having children, fear of passing on NF1 to children)

No effect or positive effect □0

Small negative effect □1

Moderate negative effect □2

Large negative effect □3

Q14 Do you suffer currently from depression or anxiety?

No problem with anxiety or depression □0

Mild anxiety or depression but can carry out my activities □1

Moderate anxiety or depression that interferes with my activities □2

Severe anxiety or depression that stops me doing my activities □3

If there is anything else you would like to add about the impact of Neurofibromatosis 1 on your quality of life please write down your comments here.
